# Loss of p53 in mesenchymal stem cells promotes alteration of bone remodeling through negative regulation of osteoprotegerin

**DOI:** 10.1038/s41418-020-0590-4

**Published:** 2020-07-21

**Authors:** Tania Velletri, Yin Huang, Yu Wang, Qing Li, Mingyuan Hu, Ningxia Xie, Qian Yang, Xiaodong Chen, Qing Chen, Peishun Shou, Yurun Gan, Eleonora Candi, Margherita Annicchiarico-Petruzzelli, Massimiliano Agostini, Huilin Yang, Gerry Melino, Yufang Shi, Ying Wang

**Affiliations:** 1 Key Laboratory of Tissue Microenvironment and Tumor, Shanghai Institute of Nutrition and Health, Shanghai Institutes for Biological Sciences, University of Chinese Academy of Sciences, Chinese Academy of Sciences, Shanghai, 200031 China; 2 Department of Experimental Medicine, TOR, University of Rome Tor Vergata, Rome, Italy; 3 Affiliated Cancer Hospital & Institute, Guangzhou Medical University, Guangzhou, 510000 China; 4 Biochemistry Laboratory, Istituto Dermopatico Immacolata (IDI-IRCCS), 00100 Rome, Italy; 5 The First Affiliated Hospital of Soochow University and State Key Laboratory of Radiation Medicine and Protection, Institutes for Translational Medicine, Soochow University, 199 Renai Road, Suzhou, 215123 Jiangsu China; 6 Medical Research Council, Toxicology Unit, University of Cambridge, Cambridge, CB2 1QP UK; 7 Present Address: Department of Experimental Oncology, Department of Experimental Oncology, IEO, European Institute of Oncology, IEO, IRCCS, 20139 Milan, Italy

**Keywords:** Cell biology, Pathogenesis

## Abstract

p53 plays a pivotal role in controlling the differentiation of mesenchymal stem cells (MSCs) by regulating genes involved in cell cycle and early steps of differentiation process. In the context of osteogenic differentiation of MSCs and bone homeostasis, the osteoprotegerin/receptor activator of NF-κB ligand/receptor activator of NF-κB (OPG/RANKL/RANK) axis is a critical signaling pathway. The absence or loss of function of p53 has been implicated in aberrant osteogenic differentiation of MSCs that results in higher bone formation versus erosion, leading to an unbalanced bone remodeling. Here, we show by microCT that mice with p53 deletion systemically or specifically in mesenchymal cells possess significantly higher bone density than their respective littermate controls. There is a negative correlation between p53 and OPG both in vivo by analysis of serum from p53^+/+^, p53^+/−^, and p53^−/−^ mice and in vitro by p53 knockdown and ChIP assay in MSCs. Notably, high expression of *Opg* or its combination with low level of p53 are prominent features in clinical cancer lesion of osteosarcoma and prostate cancer respectively, which correlate with poor survival. Intra-bone marrow injection of prostate cancer cells, together with androgen can suppress p53 expression and enhance local *Opg* expression, leading to an enhancement of bone density. Our results support the notion that MSCs, as osteoblast progenitor cells and one major component of bone microenvironment, represent a cellular source of OPG, whose amount is regulated by the p53 status. It also highlights a key role for the p53-OPG axis in regulating the cancer associated bone remodeling.

## Introduction

Bone remodeling is an exquisitely coordinated physiological process that guarantees a balance between bone formation and bone degradation to replace old or damaged bone. This process is orchestrated by cellular and molecular mechanisms occurring in osteoblasts, cells generating bone, and osteoclasts, cells degrading bone. To ensure a balanced remodeling, it is necessary that osteoblasts and osteoclasts generate and resorb the same amount of bone, phenomena indicated as “coupling” [1, 2].

This process is driven by bone morphogenetic proteins that after binding to their specific receptors activate runt-related transcription factor 2 (RUNX2), a master gene in regulating the complete differentiation of mesenchymal stem cells (MSCs) toward osteoblasts. Another important pathway regulating osteogenic differentiation of MSCs is the WNT-Frizzled-β-catenin pathway [3]. The WNT pathway can be inhibited by distinct molecules such as Dickkopf-related protein 1 secreted by various cell types in normal tissues and cytokines-activated malignant cells which can impair osteoblasts functionality [4]. Osteoclasts arise from hematopoietic stem cells that firstly differentiate toward the monocyte/macrophage lineage through the macrophage-colony stimulating factor (M-CSF). Next step involves the activation of the receptor activator of nuclear factor-κB ligand (RANKL). The binding of RANKL to RANK will start different signal transduction pathways by tumor necrosis factor (TNF) receptor-associated factor 6, nuclear factor-κB (NF-κb), and c-Jun-N terminal kinase (JNK/C Jun/fos).

Of note, a common and close niche in the bone marrow has been proposed for both osteoblasts and osteoclasts meaning that they can strictly influence and regulate each other by direct cell contact or by other mechanisms [5, 6]. The OPG/RANKL/RANK axis has been found to be crucial for bone remodeling [1, 7]. OPG is a member of the TNF receptor family which acts as a soluble decoy receptor to inhibit osteoclastogenesis [8]. It can suppress the activity and survival of osteoclasts through sequestering RANKL [9, 10] from binding to RANK on the surface of osteoclast cells. In addition, OPG was recently found to be highly expressed in advanced prostate cancer patients with bone metastasis, confirming a role in tumor progression [11]. Deletion of OPG results in a severe osteoporosis and vascular calcification due to enhanced bone resorption [12]. Oppositely, osteoporotic mice (op/op) reveal a loss of progenitor cells for both osteoclasts and macrophages with higher bone density [13].

In this context, the transcription factor p53, a well-known regulator of DNA damage response and cell death [14–20] plays a determinant role as a negative regulator of osteogenesis and osteoblast differentiation [21, 22]. Indeed, while p53 is a powerful regulator of cell death, a crucial mechanism for its role in cancer development [23–26], it has a basic effect on metabolism [27, 28] and other biologic processes [28, 29] including the regulation of MSCs [30, 31]. It is evident that cancer cells can selectively silence p53 activities in cancer stromal cells, thereby molding the cancer stroma to facilitate cancer progression [32, 33]. Indeed, inactivation of p53 and retinoblastoma gene in bone marrow derived MSCs can promote the development of osteosarcoma-like cancer when transplanted in immunodeficient mice [34]. Despite recent advances, the functions and potential molecular regulations of p53 in MSC biology during cancer progression are not fully understood.

Here, we hypothesize that there are unexplored main factors potentially contributing to the osteosclerotic-like phenotype observed in p53^−/−^ mice including: (i) p53 regulation of the molecular secretion profile of MSCs, (ii) correlation of p53 and the OPG/RANKL axis in MSCs, and (iii) p53 regulation of monocytes differentiation towards osteoclasts. By the selective deletion of p53 in mesenchymal cells, we show that p53 negatively regulates the expression of OPG in MSCs both in vitro and in vivo. More importantly, high level of *OPG* is correlated with a poor prognostic in both prostate cancer and osteosarcoma. In a prostate cancer metastasis model, impaired p53 expression can promote *Opg* expression, and enhance bone density. Overall, we speculate that p53 constrains significantly the differentiation potential of osteoclast progenitor cells toward osteoclasts, and constructs the metastatic niche of prostate cancer through the regulation of OPG expression.

## Results

### Loss of p53 impairs bone homeostasis and OPG/RANKL ratio

**Fig. 1:**
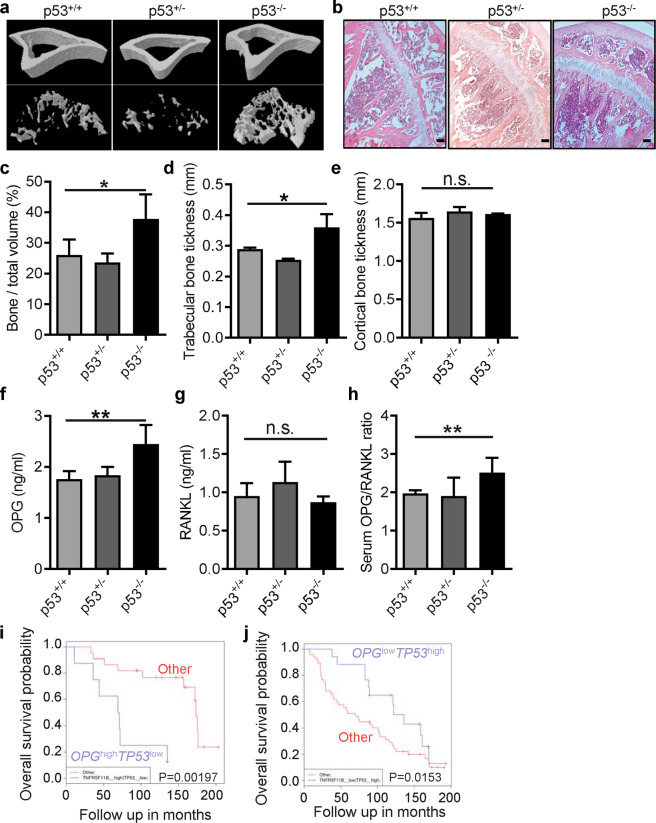
Loss of p53 impairs bone homeostasis and the normal OPG/RANKL ratio. Representative images of microCT (**a**) and H&E staining (**b**) of bone from p53^+/+^, p53^+/−^, and p53^−/−^ mice. Scale bar represents 100 µm, *n* = 5. Quantification of bone volume relative to total volume (**c**), trabecular bone thickness (**d**), and cortical bone thickness (**e**) from representative microCT scans in **a**, *n* = 5. Serum OPG (**f**) and RANKL (**g**) were measured in p53^−/−^ (*n* = 6), p53^+/−^ (*n* = 6), p53^+/+^ (*n* = 6) mice. **h** RANKL/OPG ratio was calculated according the serum values of OPG and RANKL obtained in **f** and **g**. **i**, **j** Overall survival of prostate cancer patients was analyzed by Synergy2G. GSE16560 was selected. 30 samples from batch 6 (**i**) and 64 samples from batch 4 (**j**) were shown as plots. *OPG*^high^*TP53*^*l*ow^ or *OPG*^low^*TP53*^high^ were shown as blue, and the others were shown as red. Data are presented as mean ± SD. *p < 0.05, **p < 0.01, n.s. not significant. All replicates are biological replicates.

p53 inactivation induced by mutations occurs in more than half of all cancer patients including prostate cancer and osteosarcoma [32]. The progression of these cancers is always accompanied by aberrant bone remodeling [35]. Considering the tight interplay between p53 and the process of bone homeostasis [36], we decided to investigate the link between p53 and bone biology in vivo. To do so, we analyzed both femurs and tibias harvested from mice with different p53 status by micro computed tomography (microCT) (Fig. 1a). As previously demonstrated [21], quantitative analysis revealed a significant increase in bone density in p53^−/−^ mice as compared to p53^+/+^ and p53^+/−^ control littermates (Fig. 1c). Notably, a significant increase in trabecular thickness was found in p53^−/−^ mice compared to p53^+/+^ controls (Fig. 1d), whereas no significant difference was observed in cortical bone (Fig. 1e). Histologically, haematoxylin and eosin staining performed on decalcified bone sections of p53^+/+^, p53 ^+/−^, and p53^−/−^ mice verified the results of the microCT (Fig. 1b). Because the OPG/RANKL axis plays a key role in bone remodeling and their ratio is a measure of osteoclast activation [37], we aimed to investigate their interplay with the p53 status. Serum from p53^+/+^, p53^+/−^, and p53^−/−^ mice was collected and protein levels of OPG and RANKL were assessed by the enzyme linked immunosorbent assay (ELISA). We found a significantly higher level of OPG in the serum of p53^−/−^ mice compared to p53^+/+^ ones (Fig. 1f). On the other side, no significant difference was observed for the concentration of RANKL (Fig. 1g). Furthermore, a significant increase in the OPG/RANKL ratio was found in the serum of p53^−/−^ mice, suggesting an impaired osteoclasts activity in the absence of p53 (Fig. 1h).

Assuming that the OPG/RANKL ratio is a measure of osteoclasts activation and that it has been negatively associated with the progression of bone degradation in an independent mechanism from inflammation [38], we injected RANKL subcutaneously over the femur of wild-type and p53^−/−^ mice according to an established protocol [39] in order to evaluate whether: (i) it could affect OPG/RANKL ratio, or (ii) alteration of the OPG/RANKL ratio could affect bone formation. Interestingly, we found a significant decrease of trabecular thickness and a slight decrease of bone/total volume in p53^−/−^ mice injected with RANKL compared to the control mice (Fig. S1a, b).

To reveal the role of p53 and OPG in cancer associated bone remodeling, we use Synergy2G (www.bioprofiling.de/synergy2g) to explore their correlation in prostate cancer patients [40, 41]. In GSE16560, *OPG*^high^*TP53*^low^ patients showed poor prognosis while *OPG*^low^*TP53*^high^ patients showed better overall survival, indicating a potential relationship between p53 and OPG in determination of the progression of prostate cancer (Fig. 1i, j). Given that the metastatic lesion in prostate cancers are prone to be osteoblastic instead of osteolytic, the correlation between *TP53*/*OPG* expression and overall survival could point to the possibility that low expression of *TP53* and high expression of *OPG* in prostate cancer would induce the enhancement of bone density and set up a metastatic niche. Also, *OPG* can serve as a potential prognostic biomarker in Ewing sarcoma. Kaplan–Meier analysis showed that low level of *OPG* is associated with higher event-free survival (Fig. S1c, d) and overall survival (Fig. S1e) in patients with mixed Ewing sarcoma and tumor Ewing sarcoma, respectively. In addition, *OPG* expression has an opposite prognostic value in patients with mixed Ewing sarcoma, however, the analysis fails to reach statistical significance (Fig. S1f). Collectively, all these data suggest that p53 could affect bone remodeling indirectly and contributing to higher bone intake, and thus abnormal p53 expression in cancer patients may regulate pathological bone remodeling and tumor progression through OPG.

### p53 deficiency alters the osteogenic differentiation of MSCs

**Fig. 2:**
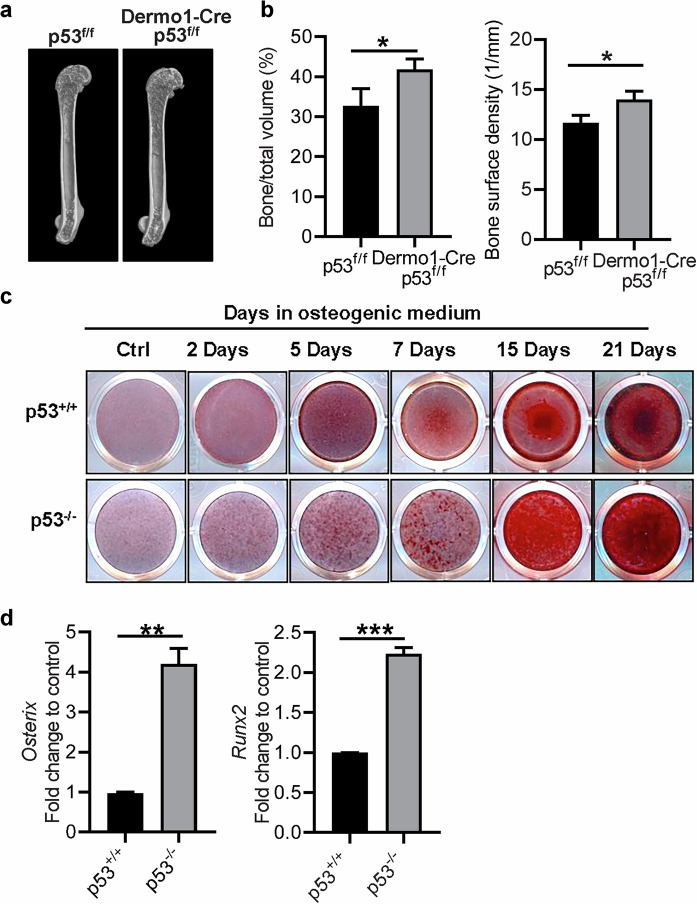
p53 deficiency alters the osteogenic differentiation of MSCs. **a** Representative microCT images from p53^f/f^ (*n* = 4) or p53^f/f^Dermo1-cre (*n* = 3) mice. **b** Quantification of bone volume relative to total volume (left) and bone surface density (right) from representative microCT scans in **a**. **c** p53^+/+^ and p53^−/−^ MSCs were cultured in osteogenic differentiation medium for indicated days and stained with Alizarin Red S. **d** p53^+/+^ and p53^−/−^ MSCs were analyzed for *Osterix*, *Runx2* mRNA to verify their basal level expression. Data are presented as mean ± SD (**b**) or mean ± SEM (**d**). *p < 0.05, **p < 0.01, ***p < 0.001. All experiments were repeated at least twice (biological replicas) with identical or comparable results.

MSCs represent a population of multipotent progenitor cells that can generate various mesenchymal lineages under specific in vitro culture condition [42, 43]. Importantly, MSCs resident in the bone marrow can assist the regeneration of mesenchymal tissues such as adipose, cartilage, and bone [44]. Therefore, according to the significantly higher bone density and trabecular bone thickness detected in p53 deficient mice, we thought to further investigate the role of p53 in bone homeostasis during osteogenic differentiation of mouse derived MSCs. To do so, we generated p53^f/f^Dermo1-cre mice that induce a specific deletion of p53 in MSCs, a cell source for generation of osteoblasts. Similar to that in p53 deficiency mice, p53^f/f^Dermo1-cre mice exhibited much higher bone density compared to that of littermate controls (Fig. 2a, b). To investigate whether p53 directly regulated MSC differentiation, we isolated MSCs from bone marrow of p53^+/+^ and p53^−/−^ mice as previously described [45]. p53 expression was shown to be undetectable in p53^−/−^ MSCs by real-time qPCR and western blotting analysis matching with the mouse genotype (Fig. S2a, b). Both p53^+/+^ and p53^−/−^ MSCs showed a common phenotype of MSCs: CD44^+^, Sca-1^+^, CD140a^+^, CD13^+^, MHC II^−^, CD11c^−^, CD11b^−^, CD86^−^, and CD45^−^ (Fig. S2c), indicating that p53 deficiency did not affect MSC phenotypic markers. To investigate whether p53 regulates osteogenesis of MSCs, we cultured p53^+/+^ and p53^−/−^ MSCs in the appropriate osteogenic differentiation medium [46]. Alizarin red staining performed at different time points of the osteogenic differentiation of MSCs revealed that p53 deficient cells exhibited an accelerated differentiation compared to wild-type MSCs (Fig. 2c). Real-time qPCR for osteogenic transcription factors involved in the early phases of osteogenic differentiation such as *Osterix* (*Osx*) and Runx2 [47] revealed their upregulation existed in p53^−/−^ MSCs compared to p53^+/+^ even without the osteogenic differentiation (Fig. 2d).

### OPG production is increased in p53 deficient MSCs

**Fig. 3:**
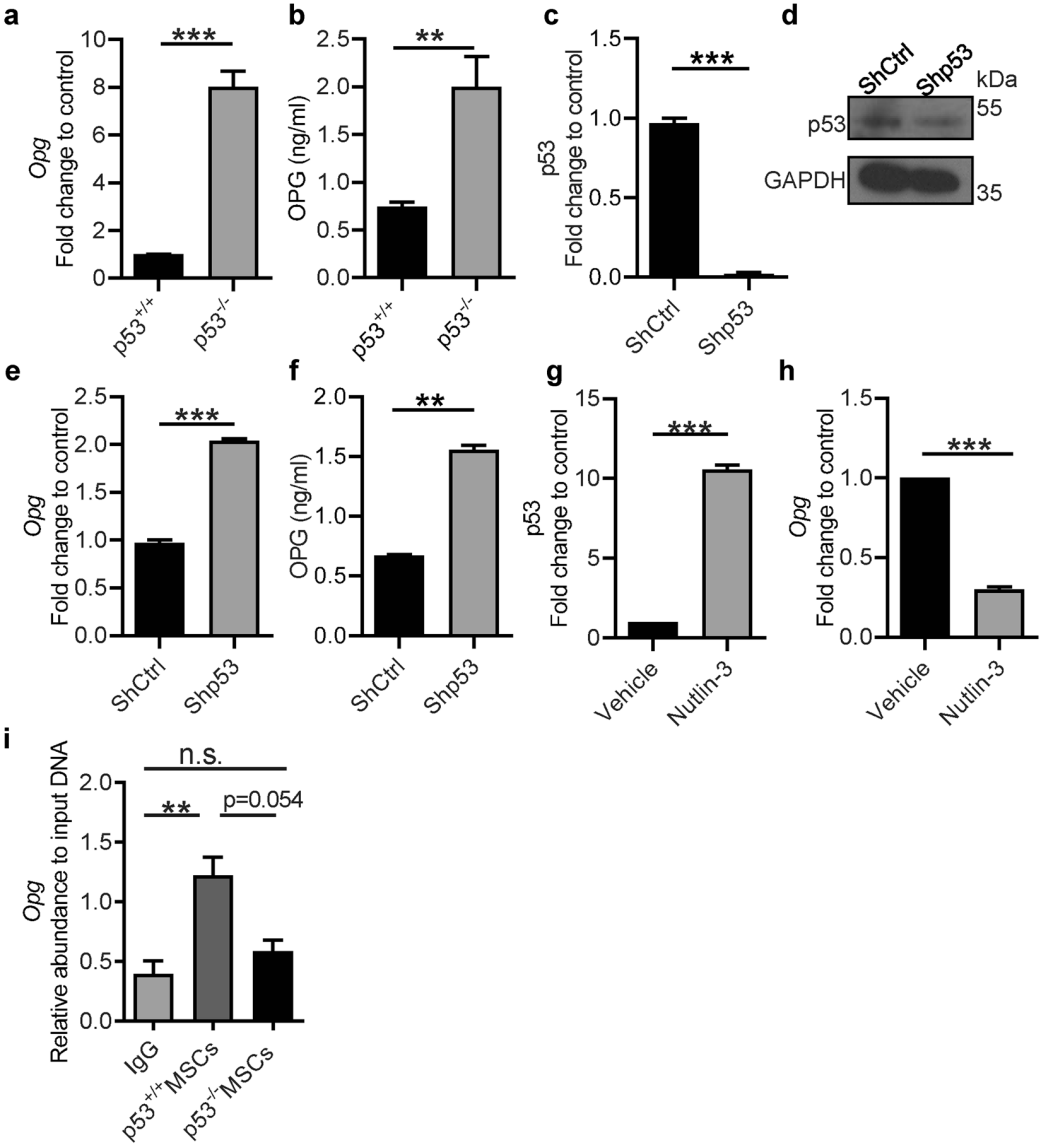
OPG production is increased in p53 deficient MSCs. **a** Quantification of *Opg* mRNA expression in p53^+/+^ and p53^−/−^ MSCs by real-time qPCR. **b** Quantification of OPG protein levels in the culture supernatant from p53^+/+^ or p53^−/−^ MSCs. **c**, **d** p53^+/+^ MSCs were transfected with p53 short hairpin RNA lentiviral particles (shp53) or control shRNA (shCtrl). After infection, cells were selected through puromycin selection (3 µg/ml) for 3 days. MSCs were analyzed for both p53 mRNA (**c**) and protein (**d**), to verify the efficiency of knockdown. **e** Quantification of *Opg* mRNA expression in shp53 and shCtrl MSCs. **f** Quantification of OPG protein levels in culture supernatants from shp53 and shCtrl MSCs. **g**, **h** Mouse bone marrow derived p53^+/+^ MSCs were treated with nutlin-3 (5 µM) or DMSO (vehicle control) for 24 h and then lysed and analyzed for p53 (**g**) and *Opg* (**h**) mRNA expression by qPCR. **i** Chromatin immunoprecipitation (ChIP) analysis was performed using anti-p53 antibodies or IgG for immunoprecipitation and subsequently analyzed with *Opg* promoter specific PCR. Data are presented as mean ± SEM. **p < 0.01, ***p < 0.001. n.s. not significant. These results are representatives of three independent experiments with similar results.

OPG is a member of the tumor necrosis factor receptor family that functions as soluble decoy receptor and inhibitor for RANKL via abrogating its interaction with RANK expressed on the surface of progenitor and mature osteoclasts [48]. However, it has not been reported whether bone marrow derived MSCs as osteoblasts precursors could represent a source for the production of OPG. For this reason, we analyzed OPG expression by real-time qPCR and ELISA in p53^+/+^ and p53^−/−^ MSCs. Compared to p53^+/+^ MSCs, p53^−/−^ MSCs exhibited higher expression and production of OPG at both mRNA and protein levels (Fig. 3a, b). These results indicate that p53 deficiency affected OPG production in MSCs, however, the changes detected in these MSCs could occur sequentially following a developmental effect of p53 loss on MSCs. To bypass this issue and to further corroborate the direct role of p53 in MSCs, we used short hairpin RNA to knock down p53 in MSCs. A successful knockdown of p53 in MSCs was demonstrated at mRNA and protein levels (Fig. 3c, d). Interestingly, we found that *Opg* mRNA and protein significantly increased in p53 knockdown MSCs (Fig. 3e, f), confirming that MSCs can act as a source for the production of OPG and p53 has a negative effect on OPG expression in MSCs.

To further investigate the negative effect of p53 on *Opg* expression in MSCs, p53^+/+^ MSCs were treated with the inhibitor Nutlin-3 to block the interaction between p53 and murine double minute 2. The upregulation of p53 mediated by Nutlin-3 treatment (Fig. 3g) repressed the expression of *Opg *in wild-type MSCs (Fig. 3h), confirming a negative regulation of OPG by p53 in MSCs. In addition, when p53 was upregulated following treatment with cisplatin to induce DNA damage (Fig. S3a–c), we observed a decrease in the *Opg* expression in p53^+/+^ MSCs (Fig. S3d). Next, we assessed whether p53 could also regulate the expression of OPG in human MSCs. To this end, we successfully knocked down p53 with shRNA in human umbilical cord derived MSCs (hUC-MSCs) as shown in Fig. S3e, f. Similar to what we observed in mouse derived MSCs, we found that the knockdown of p53 in hUC-MSCs promoted a significant increase of *OPG* expression (Fig. S3g). To test whether p53 represses the transcription of *Opg* through binding its promoter, we performed a chromatin immunoprecipitation assay (ChIP). As shown in Fig. 3i, p53 significantly associates with the *Opg* promoter in wild-type MSCs as compared to p53 deficient MSCs, suggesting that p53 represses the transcription of *Opg* by binding to the *Opg* promoter.

### Osteoclasts derived from p53^−/−^ monocytes exhibit impaired functionality

**Fig. 4:**
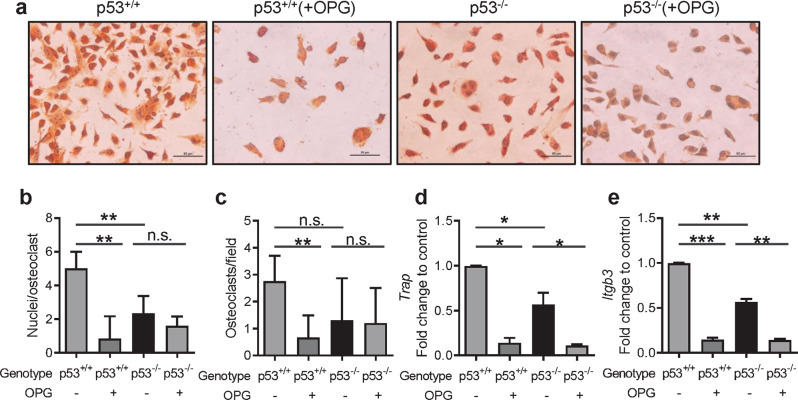
Osteoclasts derived from p53^−/−^ monocytes exhibit impaired functionality. **a** Representative images of osteoclast stained by T2RAP (red) after treatment for 7 days in specific medium plus 25 ng/ml M-CSF, 50 ng/ml RANKL, with or without OPG (50 ng/ml). Scale bar represents 50 µm. **b**, **c** Quantification of nuclei per osteoclast and TRAP ^+^ osteoclasts per field as shown in **a**. Quantification for *Trap* (**d**) and *Itgb3* (**e**) mRNA expression of experiment shown in **a**. Data are shown as mean ± SD. *p < 0.05, **p < 0.01, ***p < 0.001, n.s. not significant. The experiments were repeated at least twice (biological replicates) with similar results.

Bone remodeling is a process balanced through bone formation and bone resorption, with the latter mediated by mature and functional osteoclasts [2]. To extend the phenomena to the more general regulation of bone physiology, we assessed whether the absence of p53 could also affect osteoclast differentiation [49]. To this purpose, we isolated monocytes from bone marrow of p53^+/+^ and p53^−/−^ mice as previously described [50]. We found an increase in the percentage of Ly6C^+^ CD11b^+^ cells in p53^−/−^ mice compared to control mice (Fig. S4a, b). Then, we differentiated the isolated monocytes towards osteoclasts using conditional medium culture as previously reported [6, 50]. The differentiation of osteoclasts was investigated by tartrate resistant acid phosphatase (TRAP) staining and by real-time qPCR for specific genes expressed in mature osteoclasts. TRAP staining revealed higher percentage osteoclasts formation in monocytes derived from p53^+/+^ mice compared to that from p53^−/−^ mice (Fig. 4a–c). Along the osteoclast differentiation process, pre-osteoclasts fuse each other into mature multinucleated cells that are recognized to be active through the expression of key osteoclasts markers, such as: *Trap* and *αVβ3 integrin* chains (*β3 integrin*, *Itgb3*) [51]. We observed a significant reduction of mRNA expression of *Trap* enzyme as well as *Itgb3* in the absence of p53 (Fig. 4d, e). These results indicated that monocytes derived from p53^−/−^ mice was impaired in achieving a complete osteoclast differentiation. Moreover, in order to investigate whether in a p53 null landscape the higher expression of *Opg* from MSCs could affect osteoclast maturation, we also implemented OPG protein in the conditional medium along with RANKL and M-CSF in the cultures of monocytes as previously mentioned. In these conditions, wild-type monocytes treated with OPG were less TRAP^+^ (Fig. 4a–c) and had a significant reduction in *Trap*, *Itgb3* mRNA expression (Fig. 4d, e). Taking together, these data indicate that OPG can function as inhibitor of RANKL and induce a decreased differentiation capability in monocytes derived from wild-type mice versus osteoclasts but has less impact in the p53 null conditions.

### Regulation of the p53/OPG axis by androgen promotes bone remodeling in prostate cancer mice

**Fig. 5:**
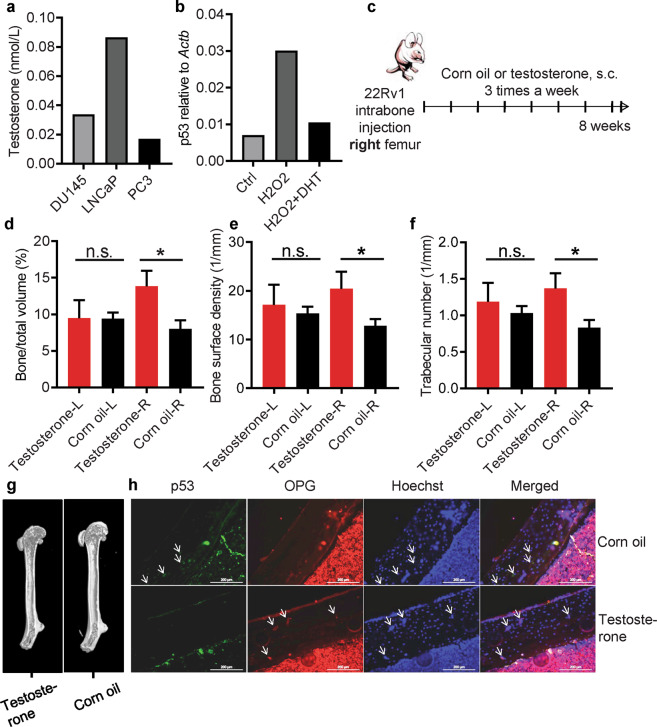
Regulation on the p53/OPG axis by androgen promotes bone remodeling in prostate cancer. **a** Testosterone concentrations in the culture supernatant from prostate cancer cell lines (DU145, LNCaP, and PC3) were analyzed by ELISA. **b** MSCs were treated with hydrogen peroxide (2 mM) alone or combined with dihydrotestosterone (DHT, 100 nM) for 6 h. p53 mRNA level was then analyzed by qPCR. **c**–**f** Human prostate cancer cell line 22Rv1 cells were intra-bone injected to the right femur of nude mice, with s.c administration of corn oil or testosterone (200 μg/mouse) three times per week for 8 weeks. **c** Schematic diagram of the tumorigenesis model. Quantification of Bone/total volume (**d**), bone surface density (**e**), and trabecular number (**f**) of the left and right femur by microCT (*n* ≥ 4). **g** Representative 3D reconstructing image of MicroCT of right femur with the administration of either testosterone or corn oil. **h** Immunohistostaining showed p53 and OPG levels in right femur. Top line was representative image of corn oil-treated control group and bottom line showed the testosterone-treated group. p53, OPG, and Hoechst were shown as green, red, and blue, respectively. Scale bar represents 200 μm. Data are shown as mean ± SD. *p < 0.05, n.s. not significant.

Given that the negative correlation between p53 and OPG is related to prognostic of prostate cancer, we further investigated the role of the p53/OPG axis in preparing the bone metastatic niche during prostate cancer progression. We questioned if the prominent hormone acting on prostate cancer would affect p53 status and the bone homeostasis. As some prostate cancer cells acquired the ability to make testosterone [52], to this end, we firstly used ELISA to assay the testosterone production by different prostate cancer cell lines, including DU145, LNCaP, and PC3 (Fig. 5a). Among them, a comparable level was demonstrated. To reveal the effect of androgen on p53 status, dihydrotestosterone (DHT) was added to hUC-MSCs treated with hydrogen peroxide (Fig. 5b). As expected, p53 was induced with the addition of hydrogen peroxide (H_2_O_2_) and significantly suppressed in the presence of DHT. Thus, enriched androgen is one of the reasons to induce the loss of activity of p53 in MSCs. We further constructed a bone metastatic model of prostate cancer by intra-bone injection of 22Rv1 cells, a prostate cancer cell line with less androgen production. During this process, 22Rv1 cells were intra-bone injected to the right femur, with *s.c* administration of corn oil or testosterone three times per week for 8 weeks (Fig. 5c). Right femur and left femur were then analyzed by microCT. As demonstrated, the percentage of bone volume, bone surface density, and trabecular number in the right femur (with prostate cancer cell injection) were much higher in testosterone-injected mice than those of corn oil injected control mice (Fig. 5d–g). By employment of immunostaining, we found that in contrast to control group, the right femur in testosterone-injected mice showed lower level of p53 and higher level of OPG (Fig. 5h). Taken together, these results demonstrate that prostate cancer cells modulate the p53/OPG axis in MSCs and their osteogenesis via androgen, and build up the bone metastatic niche.

## Discussion

**Fig. 6:**
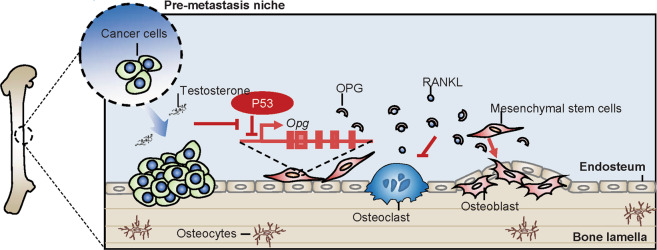
Schematic representation of the proposed molecular mechanism by which p53 regulates bone remodeling. Enriched testosterone produced by prostate cancer cells can suppress p53 expression in MSCs. These MSCs with p53 deficiency exhibit more potential to generate osteoblasts and produce OPG. The enhanced OPG production can block the RANKL-RANK signaling in osteoclasts and results in an impairment of bone resorption. Such concerted action modulated by p53 regulates the bone remodeling and constructs the bone metastatic niche of prostate cancer. OPG osteoprotegerin, RANKL receptor activator of nuclear factor κΒ ligand, RANK receptor activator of nuclear factor-κB.

Our findings revealed a strong association among p53 deficiency, high levels of OPG, and more bone formation (Fig. 6). Following the original hypothesis that osteoblasts and stromal cells could regulate osteoclasts formation, activity, and bone resorption [53, 54], OPG was reported by several groups to protect the bone from excessive bone resorption and to inhibit osteoclasts activity by binding RANKL and hence avoiding its binding to RANK receptor [55]. In keeping with the crucial role of the OPG/RANKL axis in bone biology, here we demonstrate that bone marrow derived MSCs are a source of OPG production. We found that p53 deficiency promoted a significant increment in the production of OPG at serum level and in bone marrow derived MSCs, indicating that the production of OPG from MSCs can be modulated by p53 status.

Of note, we observed a significant increase in bone density and trabecular bone thickness, but not in cortical bone in p53 null mice. This can be explained by the different anatomic location and composition of the trabeculae compared to the cortical bone. Indeed in the trabecular bone, the trabeculae are interspersed in the bone marrow that represent the bone remodeling compartment containing bone marrow progenitor cells and is invaded by blood vessels which replace hematopoietic stem cells precursors for osteoclasts [2]. On the other hand, in the cortical bone, the blood and blood elements are provided by the Haversian canals. The direct evidence that loss of p53 contributes to the development of an aberrant bone phenotype ask for new questions on the molecular mechanism by which p53 coordinates osteoblasts and osteoclasts differentiation from their respective precursor cells. It has been reported that p53 acted as a negative regulator of osteoblastogenesis through repressing the expression of *Osterix*, a transcription factor involved in the early phase of osteogenic differentiation of osteoblast precursor cells [21]. Our findings reinforce the role of p53 in the regulation of osteoblast differentiation by adding a layer of complexity. First, we showed that p53 negatively regulated the expression of *Opg* in MSCs by binding to its promoter. Previous studies have demonstrated that p53 can suppress the expression of certain genes directly or indirectly. In the context of p53 levels, cofactors or specific response element sequences used, p53 was found to repress the expression of IL-6, VEGF-A, and Osterix at the transcriptional level [21, 56–58]. The indirect repressing activity of p53 on targeted genes could be mediated by other DNA-binding proteins and co-repressors such as the CAAT-binding factor [59]. Also, its indirect regulation can be exerted by interference with the assembly of the transcription-initiation complex, or recruitment of histone deacetylase which functions as repressors of transcription [60]. Therefore, further investigation focusing on elucidating the molecular mechanism of p53 binding on *Opg* promoter will provide critical information on the detailed regulation of *Opg* expression by p53. Second, we revealed that p53 negatively regulated osteoclast differentiation. In fact, monocytes derived from p53^−/−^ mice show an impaired differentiation towards the osteoclast lineage. Moreover, secreted OPG did not have effect on the growth rate of MSCs itself (data not shown). Nevertheless, we show that the differentiation potential of p53^−/−^ monocytes into osteoclasts is also significantly impaired when we supplement OPG to the conditional medium for osteoclasts differentiation.

*Opg* deficient mice exhibit severe osteoporosis as a result from increased osteoclast activity in bone resorption, along with higher concentration of RANKL in the serum compared to wild-type mice [61]. Interestingly, we found that RANKL injection in femur of wild-type and p53^−/−^ mice reduced bone density and trabecular thickness. We performed our analysis on mice <15-week-old to avoid the presence of tumor that frequently occurs in p53 null condition and with higher frequency in p53^+/−^ mice [62]. We do not exclude the possibility that along with age, MSCs as part of the tumor microenvironment could secrete a new panel of molecules that can further affect bone homeostasis and promote tumor development or be recruited to the site of tumor formation, especially considering the influence of oxidative stress, inflammation, and tumor formation. Indeed, we provide evidence that bone marrow derived MSCs are an important source of OPG, and p53 negatively regulates its expression in prostate cancer bone metastatic niche. We suggest a link between p53 functionality in MSCs through the OPG/RANKL pathway, and we underline the importance of MSCs in regulating bone formation through their functionality and immunomodulatory properties modulated by p53. This, in the context of bone microenvironment will provide new insights related to bone remodeling and physiology but also to determine mechanisms in charge of disruption of bone homeostasis and dissociated coupling in age and in bone tumor development.

## Materials and methods

### Reagents

The following antibodies were used in flow cytometry: phycoerythrin (PE)-conjugated anti-mouse CD44 (Biolegend, 103007), Sca-I (eBioscience, 12-5981-83), CD140a (Biolegend, 135906), CD13 (BD Pharmingen, 558745), MHC II (eBioscience, 12-5320-80), CD11b (eBioscience,12-0112-83), CD11c (eBioscience, 12-0114-82), CD86 (eBioscience, 12-0862-82), and CD45 (eBioscience, 12-0451-82). Antibodies used for western blotting analysis were: anti-p53 (FL-393; sc-6243, Santa Cruz Biotechnology); GAPDH (14C10, 2118, Cell Signaling Technology). Antibody used for Chip was: p53 (FL-393; sc-6243, Santa Cruz Biotechnology), IgG (normal mouse IgG, sc2025, Santa Cruz Biotechnology). The reagents for cell treatment were: Nutlin-3 (Nutlin-3, Selleck Chemicals, Houston, TX, USA), Cisplatin (Sigma-Aldrich), RANKL (recombinant mouse RANKL, 50 ng/ml, R&D), M-CSF (recombinant mouse M-CSF, 25 ng/ml, R&D), and OPG (recombinant OPG, 50 ng/ml, Sigma-Aldrich). RANKL for mouse injection (recombinant mouse RANKL, 2 mg/kg/day) was from R&D Systems.

### Mice

C57BL/6 and nude mice were purchased from the Shanghai Laboratory Animal Center of Chinese Academy of Sciences, Shanghai, China. p53^−/−^ mice were from Animal Model Research Center of Nanjing University, Nanjing, China. Dermo1-Cre mice were purchased from The Jackson Laboratory (stock No. 008712). p53^f/f^ mice were kindly gifted by Dr. Jun Qin, Shanghai Institute of Nutrition and Health. Mice were maintained under specific pathogen-free conditions in the animal facility of Institute of Health Sciences. All procedures were approved by the Institutional Animal Care and Use Committee of the Shanghai Institute of Nutrition and Health, Shanghai Institute for Biological Sciences of Chinese Academy of Sciences.

### Cells

MSCs were derived from bone marrow of the tibia and femur of 6–8-week-old mice. The cells were cultured in DMEM medium supplemented with 10% fetal bovine serum (FBS), 2 mM glutamine, 100 U/ml penicillin, and 100 mg/ml streptomycin (all from Invitrogen, Carlsbad, CA). Non-adherent cells were discarded after 24 h, and adherent cells were maintained with medium replenished every 3 days. At confluence, cells were harvested and seeded into 96-well plates by limited dilution. Individual clones were picked and expanded. MSCs were characterized by their expression of cell surface markers and their capability to differentiate into adipocytes and osteoblasts. Cells were used before the 15th passage. Human UC–MSCs were generated as previously described [63], and approved by the institutional biomedical research ethics committee of the Shanghai Institutes for Biological Sciences (Chinese Academy of Sciences). Primary monocytes were isolated from bone marrow flushed from the tibia of 7–8-week-old wild-type and p53^−/−^ C57BL/6 mice and filtered through a 70 µm cell-strainer. Cells were washed twice in fresh new medium and plated overnight in αMEM plus 10% FBS. Prostate cancer cell lines DU145, LNCaP, PC3, and 22Rv1 were provided by Dr. Jun Qin, Shanghai Institute of Nutrition and Health.

### Differentiation of MSCs

For osteogenic differentiation, MSCs were cultured in osteogenic differentiation medium: DMEM supplemented with 10% FBS, 10 nM dexamethasone, 10 mM β-glycerophosphate, and 100 mM ascorbic acid. MSCs were seeded in 24-well plate and maintained with osteogenic medium replenished every 3 days. These cultures were stained at appropriate time points with Alizarin Red S to identify calcium deposition, an indication of osteoblasts activity. All reagents were purchased from Sigma-Aldrich (St. Louis, MO).

### Osteoclast differentiation assay

Bone marrow derived monocytes were isolated and then differentiated to osteoclasts as previously reported [6]. Briefly, 1 × 10^6^ non-adherent cells derived from overnight culture of primary monocytes were plated in 24-well plate and supplemented with 50 ng/ml RANKL and 25 ng/ml M-CSF the next day for 5 days with medium and cytokines replenishment every 2 days. An additional group for osteoclast differentiation was differentiated in the presence of 50 ng/ml OPG. Cells were fixed, TRAP was stained on day 6 using a leukocyte acid phosphatase kit (Sigma-Aldrich) and TRAP^+^ multinucleated cells were considered as mature osteoclasts.

### Histological and microCT analysis

Hind limb bones were excised from 15-week-old mice without detectable tumor, fixed with 10% neutral-buffered formalin. One limb from each mouse was collected for microCT analysis. The other hind limb was washed and decalcified in a solution of 10% EDTA for 2 weeks and then embedded in paraffin or Tissue-Tek^®^ O.C.T.™ Compound (Sakura). Paraffin sections were performed and stained with hematoxylin and eosin (H&E). For the Immunofluorescence staining, frozen sections were used and stained with anti-p53 and anti-OPG antibody following a standard protocol. Histological analysis was performed on sections of bones stained with H&E using the Zeiss Axiovert 200 microscope, and the AxioVision software version 4.6.3 SP1. MicroCT images were reconstructed and analyzed by Shanghai Showbio Biotech, Inc.

### Chromatin immunoprecipitation

A Total of 2 × 10^6^ cells were used for immunoprecipitation. The MagnaChip system (Millipore) was used according to the manufacturer’s instructions. Chromatin fragmentation was carried out by sonication of cell extract by using a Bioruptor sonicator with higher power for 30-s “on”/30-s “off” cycles for 20 min. Immunoprecipitation was performed using 8 µg anti-p53 antibody (Santa Cruz Biotechnology). Rabbit IgG were used as a negative control. DNA was purified and used as template for PCR reactions. We used primer flanking the *Opg* promoter region in mouse and in human. Primers for ChIP analysis were designed as described in a previous study [64].

### Lentiviral infection

Mouse and human p53 short hairpin RNA lentiviral particles were purchased from Santa Cruz Biotechnology (Santa Cruz, CA, USA). The lentiviral particles were added to the culture medium of MSCs and incubated for 24 h before puromycin screening. Cells were then selected through puromycin (3 µg/ml) for 3 days.

### Western blotting analysis

Cells were harvested by scraping from the culture dish and washed twice with ice-cold phosphate buffered saline (PBS). Total protein was extracted from the cell pellet with RIPA lysis buffer (Upstate, Charlottesville, VA). Samples were incubated on ice for 30 min and then centrifuged at 12,000 *g* for 20 min at 4 °C. Supernatants were collected and mixed with sodium dodecyl sulfate sample buffer, heated at 95 °C for 10 min and separated by polyacrylamide gel electrophoresis and electrical blotted to polyvinylidenedifluoride membranes (Whatman, Inc., Clifton, NJ). Membrane was incubated for 1 h at room temperature in a blocking solution composed of 5% skimmed milk powder dissolved in TBST (0.05% Tween-20, 10 mM Tris, pH 8.0, and 140 mM NaCl). After washing the membrane three times with TBST, proteins were revealed by mouse and rabbit antibodies against p53, or GAPDH by overnight incubation at 4 °C. After three washes with TBST, membranes were incubated with anti-mouse or anti-rabbit secondary antibodies (Cell Signaling Technology, Inc.). The blots were then subjected to chemiluminescent detection according to the manufacturer’s instructions.

### RNA extraction and qRT-PCR

Total RNA was extracted with TRIzol reagent (Invitrogen, Carlsbad, CA) and reverse transcribed into cDNA using the Taqman reverse transcription kit (ABI, Carlsbad, CA). The levels of mRNA of interest genes were measured by real-time qPCR (7900 HT by Applied BioSystems, Foster City, CA, USA) using SYBR Green Master Mix (TaKara Biotech). Total amount of mRNA was normalized to endogenous *Actb* mRNA. Sequences of PCR primer pairs are listed in Table 1, in Supplementary Information.

### Flow cytometry analysis

Cells were harvested and washed twice with PBS. The cell pellets were resuspended in staining buffer (PBS, 3%FBS) at a density of 1 × 10^7^ cells per milliliter. The cell suspension (100 µl) was incubated for 30 min with conjugated antibodies. Cell were washed twice with the staining buffer, and analyzed by flow cytometry through a FACS Calibur flow cytometer (Becton Dickinson, San Jose, CA). Data were analyzed through FCS Express software. At least 20,000 events were collected for each analytical point.

### ELISA immunoassay

Quantitative levels of murine OPG in the serum of p53^+/+^, p53^+/−^, and p53^−/−^ C57BL/6 mice and in the conditioned medium of MSCs were determinate in triplicate by ELISA according to the manufacturer’s protocol (Mouse OPG Quantikine ELISA kit immunoassay, R&D). Quantitative levels of murine RANKL in serum of p53^+/+^, p53^+/−^, and p53^−/−^ C57BL/6 were determined in triplicate by ELISA according to the manufacturer’s protocol (Mouse RANKL Quantikine ELISA kit immunoassay, R&D). Quantitative levels of human OPG in conditioned medium of hUC were determinate in triplicate by ELISA according to the manufacturer’s protocol (Human Osteoprotegerin TFRSF11b ELISA kit, Sigma-Aldrich). Quantitative levels of testosterone were determined following the protocol of the Testosterone Parameter Assay Kit (R&D system).

### RANKL injection

Mouse recombinant RANKL (2 mg/kg/day) or vehicle was injected subcutaneously over the femur, twice per day, for 5 days alternated by 2 days of rest. Mice were sacrificed on the 6th day, and the hind limbs were excised and processed for MicroCT analysis as already described.

### Prostate cancer model

Human prostate cancer cell line 22Rv1 cells were intra-bone injected to the right femur, with s.c administration of corn oil or testosterone (200 μg/mouse) three times per week for 8 weeks.

### Bioinformatic analysis

Bioinformatic analysis was carried out by using the following website: R2: Genomics Analysis and Visualization Platform, https://r2.amc.nl. Adjustable settings: cutoff modus: scan; Sample Filter: NO.

Overall survival of prostate cancer patients was analyzed by Synergy2G at the website www.bioprofiling.de/synergy2g. GSE16560 was selected. A total of 30 samples from batch 6 and 64 samples from batch 4 were used.

### Statistical analysis

The GraphPad Prism 5.0 software (GraphPad software, Inc., San Diego, CA) was used for the statistical analysis. Statistical significance was assessed by unpaired two-tailed Student’s test and stated as follow: *n.s*., not significant; *p  < 0.05*;* **p < 0.01*;* ***p < 0.001.

Sample size was estimated empirically, according to the exploratory experiments and published literatures with similar methodology. Since no suggestive analysis was involved in this study, randomization, and blinding were not applied.

## Supplementary information

Supplementary materials

Supplementary Figure S1

Supplementary Figure S2

Supplementary Figure S3

Supplementary Figure S4
