# Supplementary material for: Loss of p53 in mesenchymal stem cells promotes alteration of bone remodeling through negative regulation of osteoprotegerin
Source: Cell Death Differ. 2020 Jul 21;28(1):156–69. doi: 10.1038/s41418-020-0590-4 (PMC7853126; doi:10.1038/s41418-020-0590-4)
Supplement: Supplementary file 1 — Supplementary materials [file 41418_2020_590_MOESM1_ESM.docx]

**Supplementary Information for**

**Loss of p53 in mesenchymal stem cells promotes alteration of bone remodeling through negative regulation of osteoprotegerin**

Tania Velletri^1,†,#^, Yin Huang^1, #^, Yu Wang^1,#^, Qing Li^1^, Mingyuan Hu^1^, Ningxia Xie^2,6^, Qian Yang^1^, Xiaodong Chen^1^, Qing Chen^1^, Peishun Shou^1^, Yurun Gan^1^, Eleonora Candi^2,3^, Annicchiarico-Petruzzelli Margherita^3^, Massimiliano Agostini^2^, Huilin Yang^5^, Gerry Melino^2,4,*^, Yufang Shi^1,^^2,5, *^, Ying Wang^1, *^

^1^Key Laboratory of Tissue Microenvironment and Tumor, Shanghai Institute of Nutrition and Health, Shanghai Institutes for Biological Sciences, University of Chinese Academy of Sciences, Chinese Academy of Sciences, Shanghai 200031, China;

^2^Department of Experimental Medicine, TOR, University of Rome Tor Vergata, Rome, Italy.

^3^Biochemistry Laboratory, Istituto Dermopatico Immacolata (IDI-IRCCS), 00100 Rome, Italy.

^4^Medical Research Council, Toxicology Unit, University of Cambridge, Cambridge CB2 1QP, UK.

^5^The First Afﬁliated Hospital of Soochow University and State Key Laboratory of Radiation Medicine and Protection, Institutes for Translational Medicine, Soochow University, 199 Renai Road, Suzhou, Jiangsu 215123, China.

^6^Affiliated Cancer Hospital & Institute, Guangzhou Medical University, 510000 Guangzhou, China.

^†^Current address of present affiliation: Department of Experimental Oncology, Department of Experimental Oncology, IEO, European Institute of Oncology, IEO. IRCCS, 20139, Milan, Italy

*Corresponding authors: Gerry Melino ([gm614@mrc-tox.cam.ac.uk](mailto:gm614@mrc-tox.cam.ac.uk)), Yufang Shi ([yufangshi@sibs.ac.cn](mailto:yufangshi@sibs.ac.cn)) and Ying Wang **(**[yingwang@sibs.ac.cn](mailto:yingwang@sibs.ac.cn))

^#^These authors contributed equally to this work.

**This PDF file includes:**

Supplementary Figure Legends and Table

**Supplementary figures Legends**

**Figure S1 related to Figure 1:**

(A) Quantification of trabecular bone thickness from microCT scans. n=4. (B) Quantification of bone volume relative to total volume from micro-CT scans. n=4. (C-E) Low *Opg* mRNA levels were associated with better prognosis of patients with Ewing sarcoma. (F) Kaplan Meier analysis in patients with mixed sarcoma according with *Opg* mRNA levels. Data are shown as mean ± SD. * p<0.05, ** p<0.01, *** p<0.001, n.s. not significant.

**Figure S2 related to Figure 2:**

(A) Quantification of p53 mRNA expression by real-time qPCR of p53^+/+^, p53^+/-^ and p53^-/-^ MSCs. Data are shown as mean ± SEM. *** p<0.001. (B) Quantification of p53 protein levels in p53^+/+^, p53^+/-^ and p53^-/-^ MSCs by western blot. (C) MSCs surface markers were analyzed by flow cytometry. p53^+/+^ and p53^-/-^ MSCs were stained with the indicated antibodies or isotype controls and analyzed by flow cytometry. Data were analyzed by FCS Express software. Areas in grey shadow represent isotype controls; red lines represent specific antibodies.

**Figure S3 related to Figure 3:**

(A) Annexin V and propidium iodide double staining of p53^+/+^ MSCs treated with the vehicle dimethyl sulfoxide (DMSO), or with cisplatin. Data were analysed by FCS-Express software. (B) Quantification of p53 mRNA expression in p53^+/+^ MSCs treated with cisplatin for 48 hours. The experiments were repeated twice. (C) Quatification of p53 protein levels in p53^+/+^ MSCs after cisplatin treatment. (D) *Opg* mRNA expression in p53^+/+^ MSCs treated with cisplatin for 48h. (E and F) Human umbilical cord derived MSCs were infected by p53 shRNA (shp53) lentiviral particles or control shRNA lentiviral particles (shCtrl) for 24 hours. Puromycin was then added to the culture at the concentration of (3 μg/ml) after removal of lentiviral particles. Cells were harvested and collected after 3 days for the detection of p53 at mRNA (E) and protein level (F). (G) OPG mRNA quantification after silencing p53 in human umbilical cord derived MSCs. Data are shown as mean ± SEM. * p<0.05, *** p<0.001. The experiments were repeated at least twice (biological replicates) with similar results.

**Figure S4 related to Figure 4:**

(A) Flow cytometry analysis of bone marrow derived monocyte subset from p53^+/+^ or p53^-/-^ mice. Cells were analyzed by antibodies to CD11b and Ly6C. (B) Representative percentage of CD11b^+^ and Ly6C^+^ monocytes as described in (A).

**Supplementary Table 1. Primers for quantitative real time PCR.**

| **Primers (mouse)** | **Sequence** |
| --- | --- |
| **p53 forward** | **5’-GTCACAGCACATGACGGAGG-3’** |
| **p53 reverse** | **5’-****TCTTCCAGATACTCGGGATAC-3’** |
| ***Opg* forward** | **5’-ACCCAGAAACTGGTCATCAGC-3’** |
| ***Opg* reverse** | **5’-CTGCAATACACACACTCATCACT-3’** |
| ***Runx2* forward** | **5’- CCACGGCCCTCCCTGAACTCT -3’** |
| ***Runx2* reverse** | **5’- ACTGGCGGGGTGTAGGTAAAGGTG -3’** |
| ***Osterix* forward** | **5’- ATGGCGTCCTCTCTGCTTG -3’** |
| ***Osterix* reverse** | **5’- TGAAAGGTCAGCGTATGGCTT -3’** |
| ***Trap* forward** | **5’-CACTCCCACCCTGAGATTTGT-3’** |
| ***Trap* reverse** | **5’-CATCGTCTGCACGGTTCTG-3’** |
| ***Itgb3* forward** | **5’-CCACACGAGGCGTGAACTC-3’** |
| ***Itgb3* reverse** | **5’-CTTCAGGTTACATCGGGGTGA-3’** |
| ***Actb* forwards** | **5’-TTCCAGCCTTCCTTCTTGGG-3’** |
| ***Actb* reverse** | **5’-TGTTGGCATAGAGGTCTTTACGG-3’** |

| **Primers (human)** | **Sequence** |
| --- | --- |
| **p53 forward** | **5’-CCCCTCCTGGCCCCTGTCATCTTC-3’** |
| **p53 reverse** | **5’-GCAGCGCCTCACAACCTCCGTCAT-3’** |
| ***OPG* forward** | **5’-GCGCTCGTGTTTCTGGACA-3** |
| ***OPG* reverse** | **5’-AGTATAGACACTCGTCACTGGTG-3’** |
| ***ACTB* forward** | **5’-GGACTTCGAGCAAGAGATGG-3’** |
| ***ACTB* reverse** | **5’-CACCTTCACCGTTCCAGTTT-3’** |
