# Supplementary figures and images for: Loss of p53 in mesenchymal stem cells promotes alteration of bone remodeling through negative regulation of osteoprotegerin
Source: Cell Death Differ. 2020 Jul 21;28(1):156–69. doi: 10.1038/s41418-020-0590-4 (PMC7853126; doi:10.1038/s41418-020-0590-4)

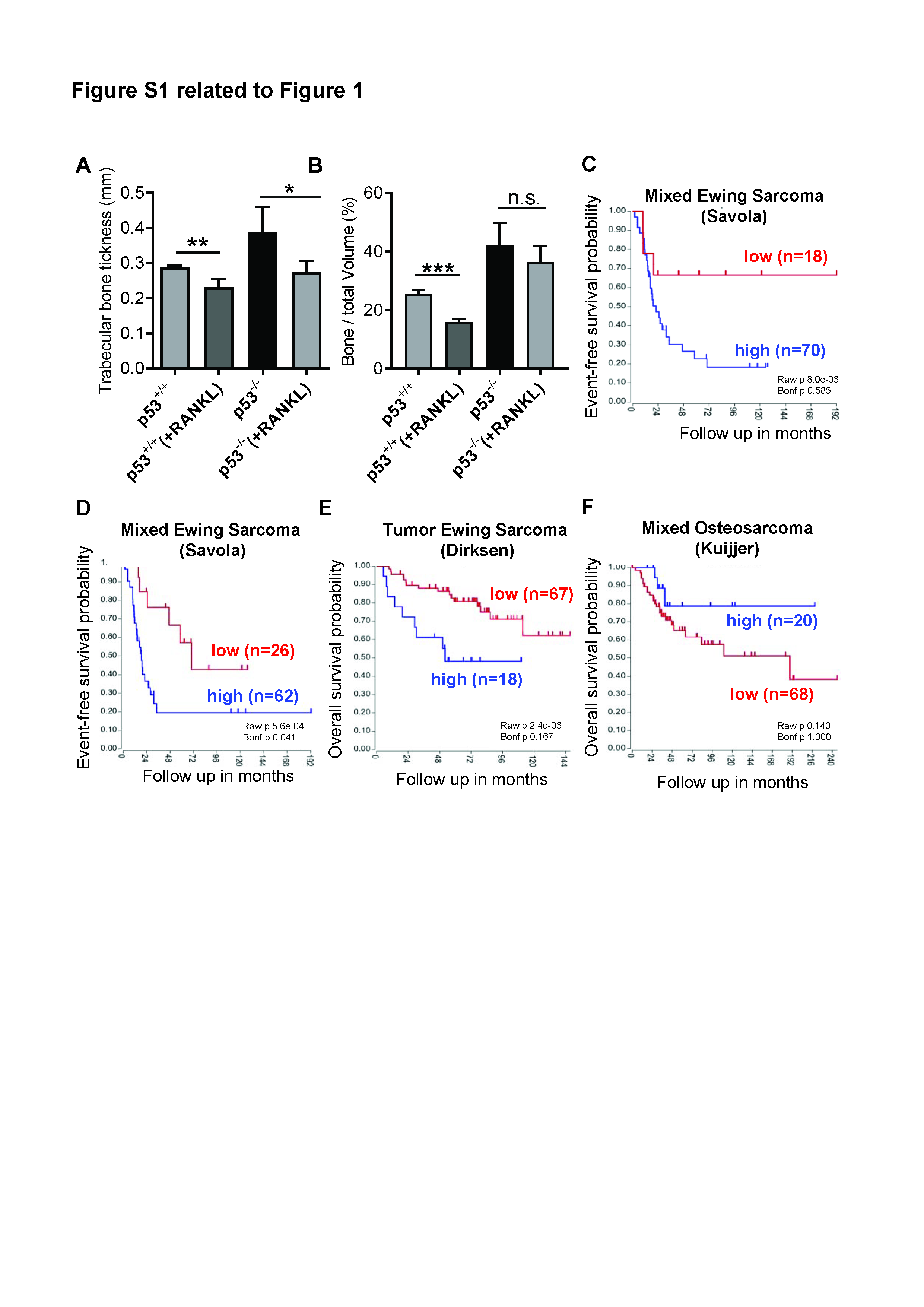

Supplement: Supplementary file 2 — Supplementary Figure S1 [file 41418_2020_590_MOESM2_ESM.tif]

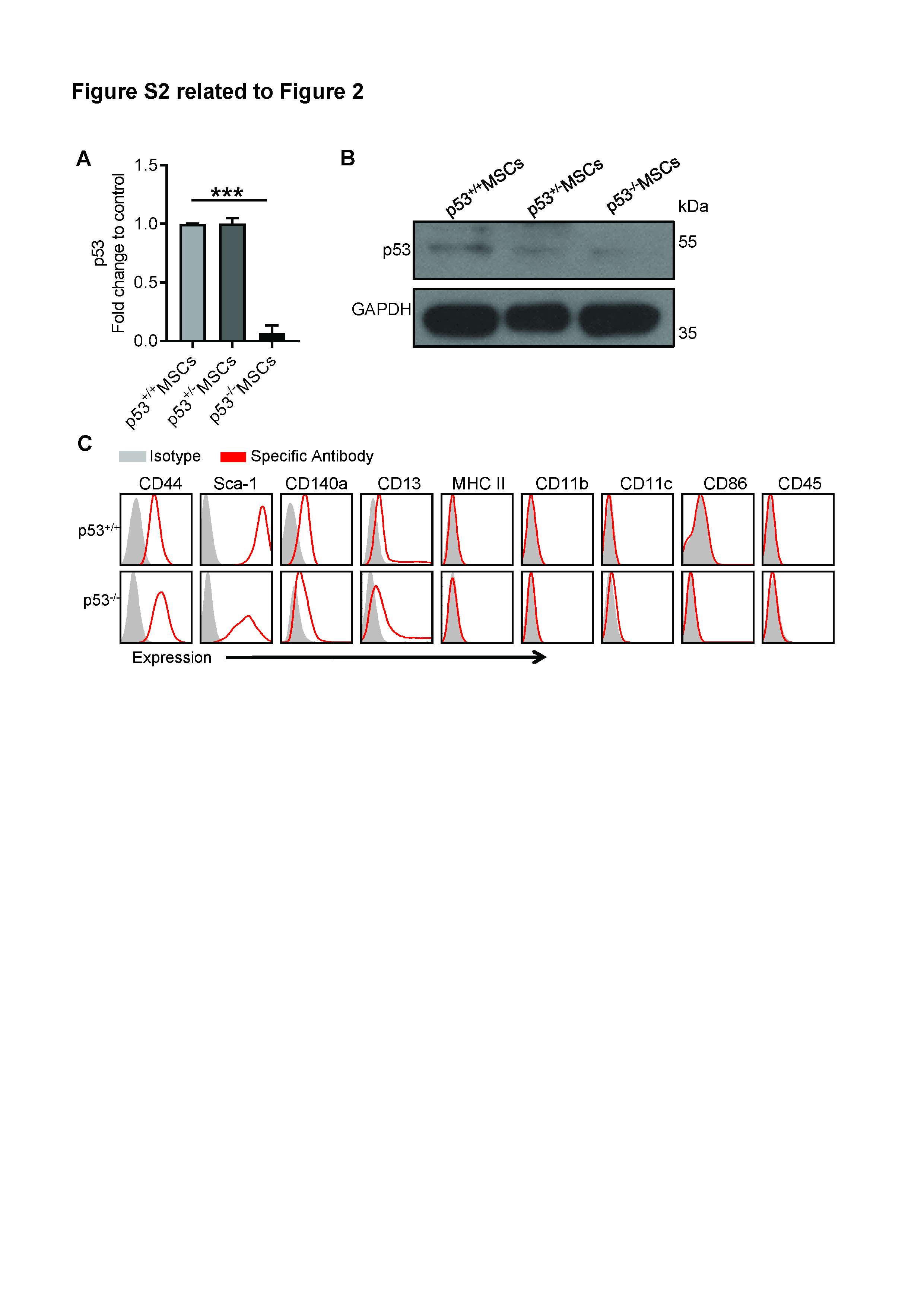

Supplement: Supplementary file 3 — Supplementary Figure S2 [file 41418_2020_590_MOESM3_ESM.tif]

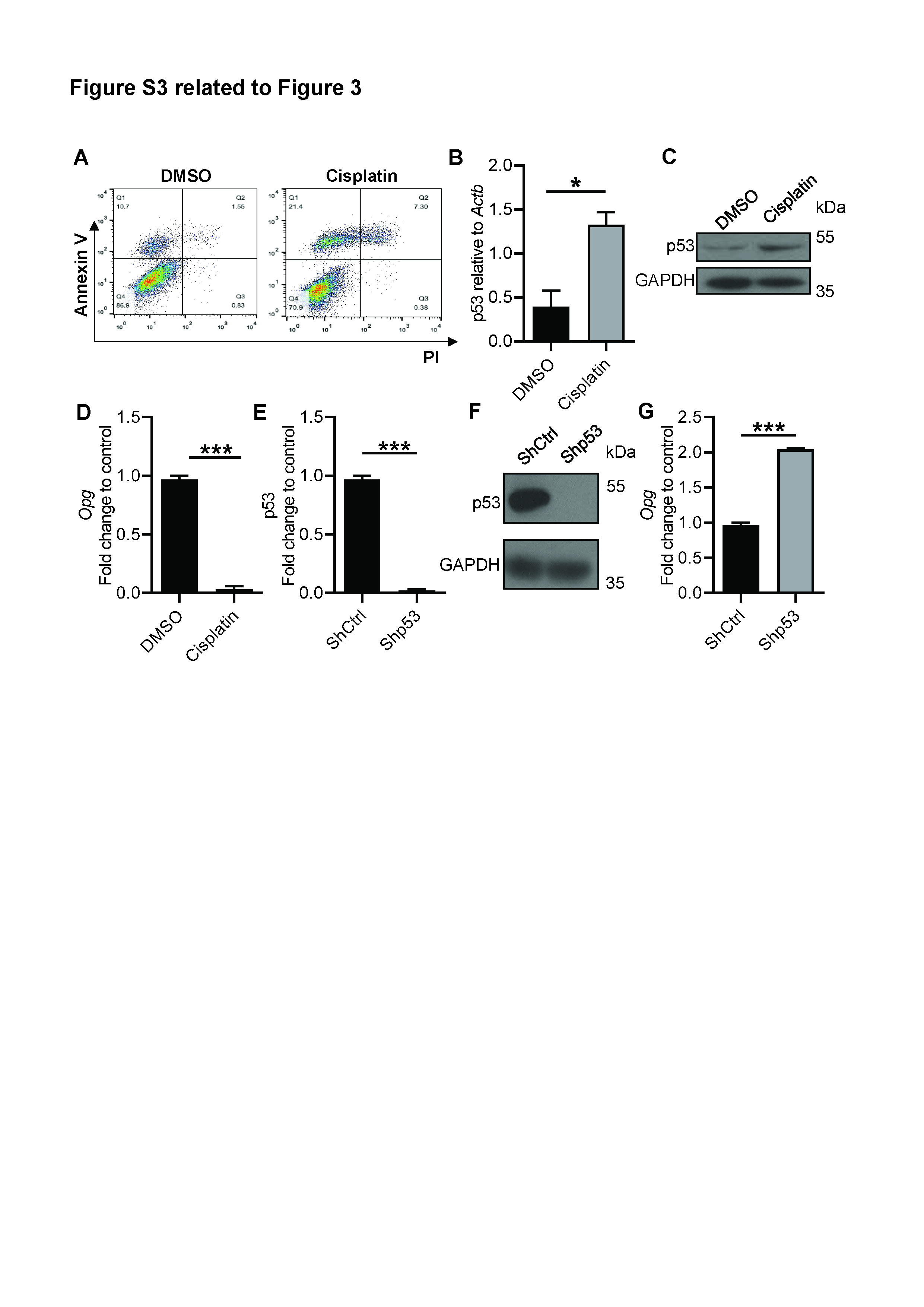

Supplement: Supplementary file 4 — Supplementary Figure S3 [file 41418_2020_590_MOESM4_ESM.tif]

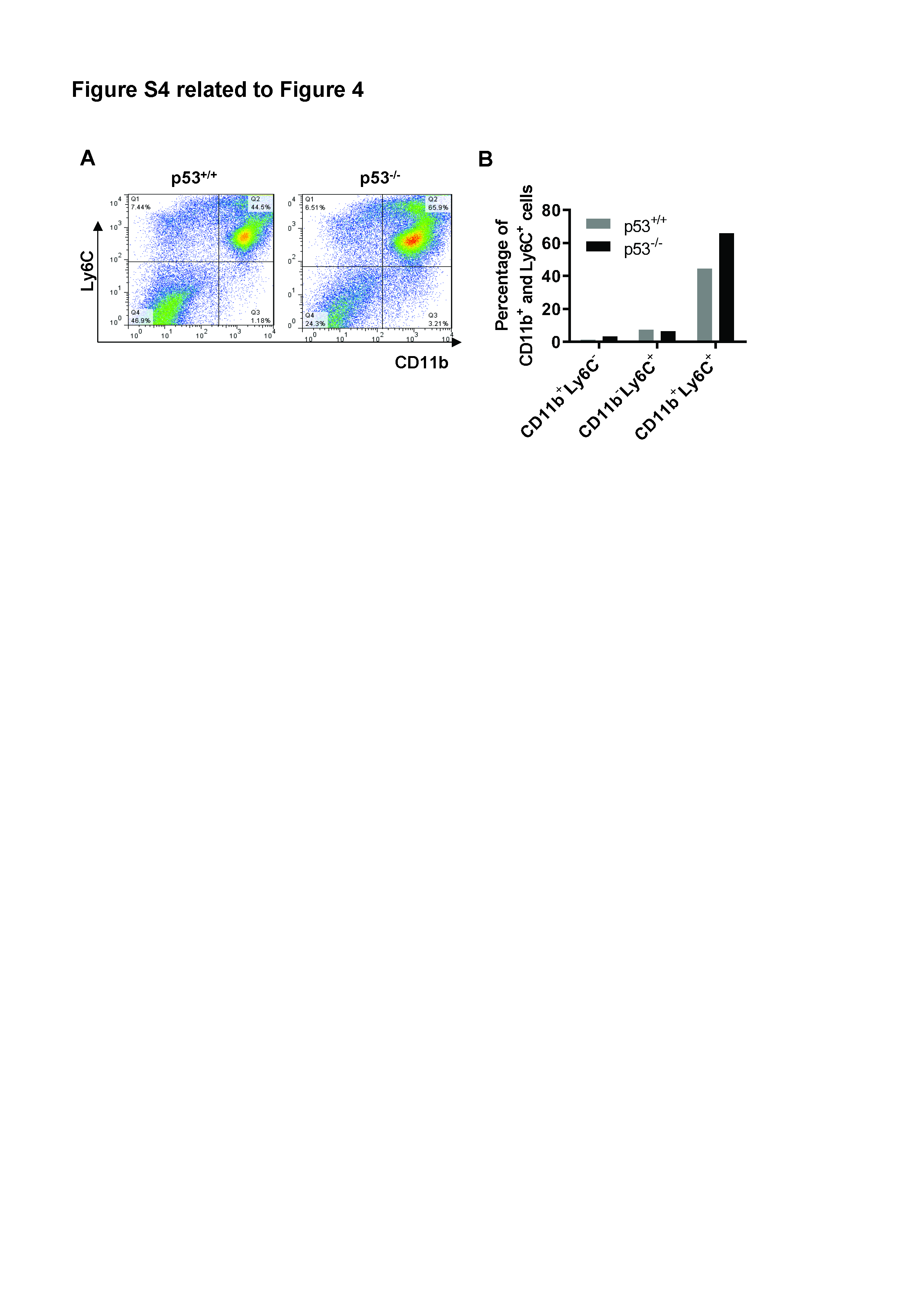

Supplement: Supplementary file 5 — Supplementary Figure S4 [file 41418_2020_590_MOESM5_ESM.tif]
